# Factors affecting Malaysian ESL teachers' behavioral intentions for technology use in the post-COVID-19 era

**DOI:** 10.3389/fpsyg.2023.1127272

**Published:** 2023-03-22

**Authors:** Teo Woon Chun, Melor Md Yunus

**Affiliations:** ^1^Sekolah Jenis Kebangsaan (Cina) Peay Min, Pengerang, Johor, Malaysia; ^2^Faculty of Education, Universiti Kebangsaan Malaysia, Bangi, Selangor, Malaysia

**Keywords:** English as a second language, technology, behavioral intention, Malaysian ESL teachers, post-COVID-19 era, UTAUT model, education

## Abstract

This study aimed to investigate English as a Second Language (ESL) teachers' technology acceptance levels and to identify the factors affecting their behavioral intentions (BI) with respect to technology use in the post-COVID-19 era. A cross-sectional survey of 361 Malaysian ESL teachers was conducted. Participants were recruited *via* convenience sampling, and they answered an online survey questionnaire that was designed with reference to past studies. The collected data were analyzed *via* descriptive statistics, Pearson's correlation, and multiple regression analyses. The findings revealed that Malaysian ESL teachers generally had a high level of technology acceptance in the post-COVID-19 era. Their BIs had a significant relationship with three factors: performance expectancy (PE), effort expectancy (EE), and social influence (SI), of which EE was identified as the most significant factor influencing their BI with respect to technology use in the post-COVID-19 era. Conversely, the presence of facilitating conditions did not have a substantial connection with ESL teachers' behavioral intentions for technology use after the pandemic, despite the fact that there was weak positive relationship with each other. This study provides insights for the field of educational psychology by identifying the current trends in ESL teachers' behavioral intentions in adopting technology in the post-COVID-19-era ESL classrooms. The findings of this study may also support investigations into technology acceptance in ESL teaching, illustrating a growing need to provide adequate educational and technological tools, resources, and facilities to facilitate the delivery of lessons by ESL teachers. Future studies should conduct longitudinal research and investigate more variables from different technology acceptance models.

## 1. Introduction

Technology continues to develop in significant ways to assist English as a Second Language (ESL) teachers in facilitating language learning for their students (Wei et al., 2023). However, the abrupt shift in the delivery mode of ESL lessons due to the emergence of the COVID-19 pandemic has resulted in widespread challenges for access to quality education, as highlighted by the fourth Sustainable Development Goal (SDG) (Rafiq et al., 2022). Accordingly, the COVID-19 pandemic forced almost all countries around the world to switch from conventional teaching methods to the alternative of fully-fledged online teaching and learning (Ye et al., 2023). Thus, the use of technology has been essential during the COVID-19 pandemic, as it was heavily relied on to provide emergency remote teaching (ERT). Consequently, ESL teachers endeavored to learn how to use various online applications and tools to deliver effective ESL lessons and to engage their pupils in an effort to avoid learning loss (Mohtar and Yunus, 2022).

During the implementation of ERT, ESL teachers had to suddenly adopt technology to deliver ESL lessons to ensure their students' continued learning, regardless of their technological competency and acceptance level. Studies on ESL teachers' adoption of technology during the pandemic have since been conducted. For instance, Li's study (2022) on high school EFL teachers in China showed that they voluntarily learned how to integrate technology for teaching purposes. Furthermore, Wen and Tan (2020) claim that the COVID-19 pandemic served as a driving force for the adoption of technology among primary and secondary ESL teachers for ERT.

As ESL teachers conducted ERT throughout the pandemic, it has been proposed that the COVID-19 pandemic has driven teachers to demonstrate their abilities in utilizing technologies with the adoption of technological infrastructure provided to maximize the benefit of technology-based education (Choi et al., 2021). Hence, ESL teachers could utilize the technological knowledge and skills acquired from ERT to improve the quality of their ESL lessons, since mediation of technology use in these lessons could potentially contribute to desired learning outcomes (Hennessy et al., 2022). Thus, it is necessary to measure ESL teachers' technology acceptance levels in order to ensure quality education as part of the SDG (Yaccob et al., 2022), particularly in primary and secondary ESL classrooms.

The Unified Theory of Acceptance and Use of Technology (UTAUT) is one of the most comprehensive models of technology acceptance; this model was developed by Venkatesh et al. (2003). It was then revised to UTAUT-2 in 2012 to develop an overarching framework for examining technology acceptance (Venkatesh et al., 2012). The UTAUT-3 framework was later introduced as an extension of the UTAUT-2 model (Farooq et al., 2017). The UTAUT model was employed in this study because of its simplicity and understandability compared to UTAUT-2 and UTAUT-3 (Awa and Ukoha, 2020). Moreover, the UTAUT model remains a well-established and validated tool for assessing technology acceptance among users across various professions and industries (Al-zboon et al., 2021; Yang et al., 2023).

**Figure 1:**
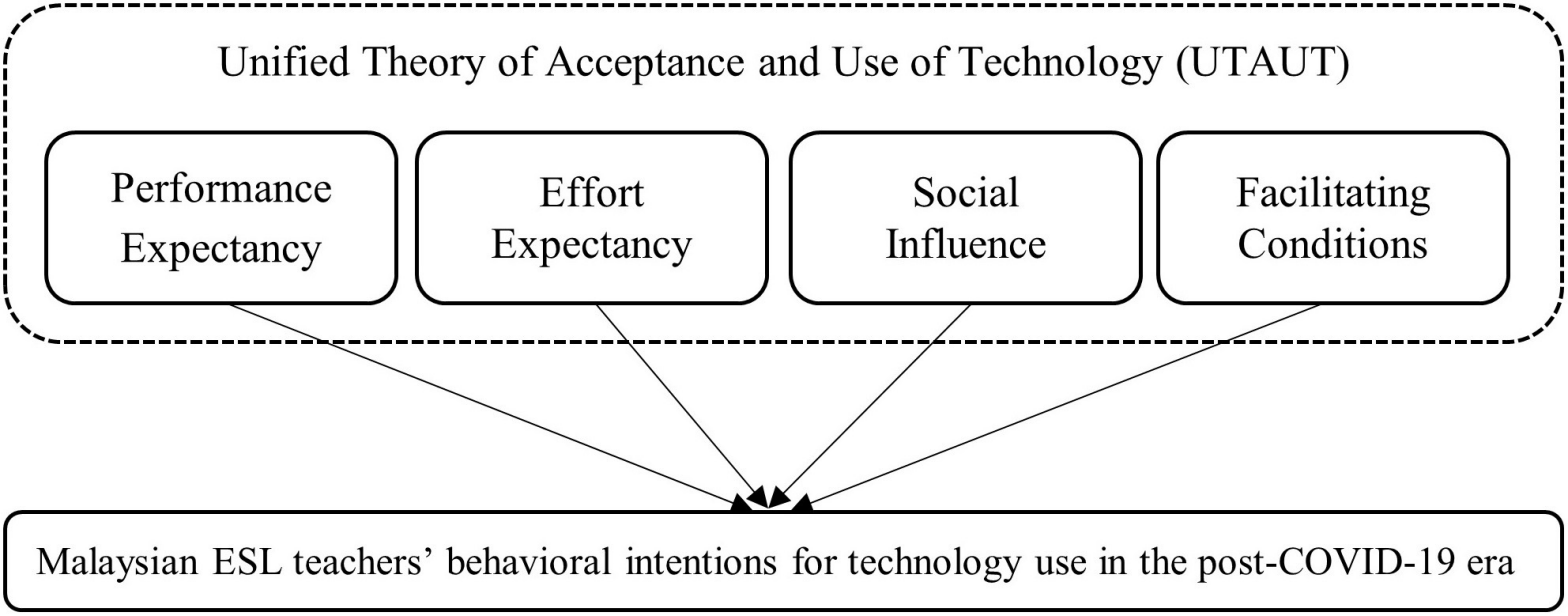
Conceptual framework of the study.

Four factors affect technology users' behavioral intentions (BI) in the UTAUT model (Venkatesh et al., 2003). Performance expectancy (PE) concerns the degree to which the technology can provide benefits and improve his or her performance to a level that is on par with expectations. Effort expectancy (EE) relates to the extent of ease of using technology. Social influence (SI) relates to the degree to which an individual perceives that it is important that others believe he or she should use the new system. Facilitating conditions (FC) represent the extent to which a technology user perceives that the existing organizational and technical support can facilitate their experience and intentions toward technology use. These four constructs have been proven to contribute to individuals' intentions toward technology use, which is relevant to this study as illustrated in Figure 1.

Nevertheless, research on changes in ESL teachers' technology acceptance levels after the COVID-19 pandemic is still deficient, leaving a gap. Therefore, ESL teachers' acceptance of technology during the COVID-19 pandemic is worth investigating to identify their intentions of using and integrating technology in their post-COVID-19 teaching and learning sessions, which will inform predictions of future educational trends and promote sustainable education aligned with the SDG (Sung et al., 2020).

Hence, this study aimed to examine ESL teachers' technology acceptance levels and factors affecting their intentions to use technology in their post-COVID-19 ESL classroom. Specifically, it sought to answer the following research questions:

1. What is the technology acceptance level of Malaysian ESL teachers in the post-COVID-19 era?
2. To what extent is there a relationship between the factors (PE, EE, SI, and FC) and Malaysian ESL teachers' behavioral intentions toward technology use in the post-COVID-19 era?
3. What is the most significant factor that influences Malaysian ESL teachers' behavioral intentions to use technology in the post-COVID-19 era?

## 2. Methods

### 2.1. Research design

This study employed a cross-sectional survey design to examine Malaysian ESL teachers' technology acceptance level and factors affecting their BI with respect to accepting the use of technology in their ESL classrooms. The UTAUT framework was used as the underlying theory of this study.

### 2.2. Research sample

A total of 361 ESL teachers from primary and secondary schools in Malaysia were recruited to participate in this study *via* the convenience sampling method. The participants who took part in the study fulfilled the pre-requisite of working in either a primary or a secondary school in Malaysia and having experienced ERT during the COVID-19 pandemic. This ensured that only eligible respondents answered the questionnaire to achieve this study's objectives (Andrade, 2020). Overall, 61 male (16.9%) and 300 female (83.1%) ESL teachers located all over Malaysia participated in this study. Most of them were 31–40 years old (37.7%), held a bachelor's degree (76.5%) in Teaching of English as a Second Language (TESL) (62.6%), and had up to 10 years of English teaching experience (66.8%) at a government primary school (81.2%) located in an urban area (46.5%) (Supplementary Table 1).

### 2.3. Research instrument

The cross-sectional survey method of data collection was employed. There were 20 items in the devised survey questionnaire based on the main five constructs of the UTAUT model, namely PE, EE, SI, FC, and BI, with reference to the relevant literature (Thompson et al., 1991; Huang et al., 2021; Mohammad-Salehi et al., 2021; Yunus et al., 2021; Ateş and Garzón, 2022). These items tended to examine the circumstances that affected the respondent's will to adopt technology, and referred to practices for facilitating ESL teaching and improving their performance by devising, adopting, and managing appropriate technological processes and resources to complete a certain task.

The questionnaire used in this study employed a 5-point Likert scale, with “strongly disagree” on one end, “strongly agree” on the other, and “neutral” in the middle. This approach was employed due to its practicality in measuring the participants' level of agreement or disagreement with a variety of statements about their attitude, which was relevant to this study (Taherdoost, 2019).

The instrument's construct validity was assessed to ensure that it was pertinent to the proposed study. Three experts specializing in related fields vetted the questionnaire. The reliability of this instrument was also examined to ensure its ability to produce similar results with repeated measurements. The items in the questionnaire each have a Cronbach's alpha coefficient between 0.74 and 0.96, which shows high reliability, as suggested by Sekaran and Bougie (2017).

### 2.4. Data collection procedure

The survey questionnaire was forwarded to Malaysian ESL teachers' social media platforms, such as Telegram, WhatsApp, and Facebook. Consent to use these platforms to disseminate the questionnaire was obtained from the administrators of the social media platforms. The teachers in the group were informed of the purpose of the survey questionnaire, and those who agreed to participate in the study were asked to follow the link provided, which then brought them to the Google Forms questionnaire. The data collection process ended after an adequate number of valid responses were received. The survey findings obtained *via* Google Forms were then transferred to Microsoft Excel and SPSS version 26.0 for data analysis.

### 2.5. Data analysis procedure

The data collected from the questionnaire were analyzed using SPSS version 26.0. Descriptive statistics, including frequencies, percentages, means, and standard deviations, were used to determine ESL teachers' technology acceptance levels. Pearson's correlation analysis was then applied to identify the relationship between the factors in the UTAUT model and their BI in the post-COVID-19 era. Multiple regression analysis was conducted to test the research hypotheses and identify the factor that has the most significant impact on ESL teachers' BI toward use of technology in the post-COVID era.

## 3. Results

### 3.1. Research question 1: What is the technology acceptance level of Malaysian ESL teachers in the post-COVID-19 era?

Considering the first objective of this study, all valid responses were examined in the form of frequencies and percentages (Supplementary Table 2). All variables in this study were also analyzed descriptively, with the mean and standard deviation calculated for each domain. The interpretation of means followed guidelines for interpretation adapted from Best and Kahn (2006), in which a mean value in the range of 1.00–2.33 was taken to indicate a low level of acceptance, 2.34–3.67 a moderate level, and 3.68–5.00 a high level of acceptance. Table 1 summarizes the computed means and standard deviations of all the items according to the relevant construct.

**Table 1:** Malaysian ESL teachers' technology acceptance levels in the post-COVID-19 era.

**Variable**	**Mean**	**Std. Deviation**	**Interpretation**
Performance expectancy (PE)	4.41	0.610	High
Effort expectancy (EE)	4.15	0.674	High
Social influence (SI)	4.12	0.659	High
Facilitating conditions (FC)	3.21	0.958	Moderate
Behavioral intention (BI)	4.32	0.669	High

According to Table 1, the means of all domains varied from 3.21 to 4.41, while the standard deviations of all variables ranged from 0.610 to 0.958, indicating a narrow spread of scores around the mean. The means of all variables (except FC) were at least 3.68, indicating that the overall technology acceptance level of the respondents is considered high.

The findings demonstrate that Malaysian ESL teachers intended to employ technology in the post-COVID-19 era because they found it useful ($\bar{\text{X}}$ = 4.41, σ = 0.610) and easy to use ($\bar{\text{X}}$ = 4.15, σ = 0.674). Additionally, the influence of the people around them positively impacted their BI ($\bar{\text{X}}$ = 4.12, σ = 0.659), resulting in a high mean for BI ($\bar{\text{X}}$ = 4.32, σ = 0.669). On the other hand, a moderate level of acceptance indicated by the mean response regarding FC ($\bar{\text{X}}$ = 3.21, σ = 0.958) implies that the respondents might adopt technology only to a moderate degree due to the lack of technology tools and resources available to conduct ESL lessons after the pandemic.

### 3.2. Research question 2: To what extent is there a relationship between the factors (PE, EE, SI, and FC) and Malaysian ESL teachers' behavioral intentions for technology use in the post-COVID-19 era?

To accomplish the second objective of this study, Pearson's correlation analysis was performed *via* SPSS version 26.0. A normality test was conducted prior to the analysis by examining the skewness and kurtosis of the data set. Both skewness and kurtosis values fell within the range of **–**1 to 1, indicating the normality and symmetry of the collected data (Orcan, 2020). Table 2 displays the Pearson correlation coefficient values of all the independent variables of this study with the dependent variable of behavioral intention.

**Table 2:** Correlation between each of the factors and the respondents' behavioral intentions to use technology in the post-COVID-19 era.

		**Performance expectancy (PE)**	**Effort expectancy (EE)**	**Social influence (SI)**	**Facilitating conditions (FC)**
Behavioral intention (BI)	Pearson correlation	0.661^**^	0.656^**^	0.585^**^	0.283^**^
	Interpretation (Mukaka, 2012)	Moderately positive	Moderately positive	Moderately positive	Negligible
Sig. (2-tailed)	0.000	0.000	0.000	0.000

** Correlation is significant at the 0.01 level (2-tailed).

The findings revealed that there are three moderately positive relationships between the factors and BI, namely those of PE (r = 0.661), EE (r = 0.656), and SI (r = 0.585). Conversely, there is a negligible relationship between FC (r = 0.283) and ESL teachers' BI. In other words, the relationship between the FC experienced by the respondents and their BI to use technology after the COVID-19 pandemic is minimal, resulting in a weak positive correlation between these variables.

The significance of the relationships between the factors in the UTAUT model and ESL teachers' BI to use technology was examined *via* multiple regression analysis. Table 3 shows the regression coefficients of each of the factors on ESL teachers' BI. The null hypotheses of this study were mainly rejected with reference to the presented findings of the current study.

**Table 3:** Regression of factors on the respondents' behavioral intentions (BI).

	**Standardized coefficient (β)**	***t*-value**	**Sig**.	**R^2^**
Performance expectancy (PE)	0.301	5.837	0.000	0.437
Effort expectancy (EE)	0.325	6.614	0.000	0.430
Social influence (SI)	0.216	4.732	0.000	0.342
Facilitating conditions (FC)	0.055	1.484	0.139	0.080

#### 3.2.1. Hypothesis 1: There is no significant relationship between PE and Malaysian ESL teachers' BI to use technology in the post-COVID-19 era

Hypothesis 1 is rejected as multiple regression analysis implies that this variable explains 43.7% of the variance in BI [R^2^ = 0.437, F (4,356) = 110.532, *p* < 0.01]. PE significantly predicts ESL teachers' BI to use technology in the post-COVID-19 era [β = 0.301, t (360) = 5.837, *p* < 0.01]. PE also has a moderately positive correlation with their BI for technology use after the pandemic [r (360) = 0.661, *p* < 0.01]. This shows that ESL teachers' BI to use technology is boosted when they expect that it can improve their job performance.

#### 3.2.2. Hypothesis 2: There is no significant relationship between EE and Malaysian ESL teachers' BI to use technology in the post-COVID-19 era

Hypothesis 2 is rejected as there is a significant regression equation in which F (4,356) = 110.532, *p* < 0.01, with an R^2^ of 0.430, indicating that 43% of the variance in BI can be explained by EE. The multiple linear regression coefficient was calculated for the prediction of BI based on EE, β = 0.325, t (360) = 6.614, *p* < 0.01. The Pearson correlation test result also proves that EE has a moderately positive association with BI to use technology in the post-COVID-19 era [r (360) = 0.656, *p* < 0.01]. This indicates that ESL teachers are keen to apply technology for instructional purposes because they find it manageable.

#### 3.2.3. Hypothesis 3: There is no significant relationship between SI and Malaysian ESL teachers' BI to use technology in the post-COVID-19 era

Hypothesis 3 is rejected as SI significantly predicts ESL teachers' BI to use technology after the COVID-19 pandemic, β = 0.216, t (360) = 4.732, *p* < 0.01. This variable explains 34.2% of the variance in BI, R^2^ = 0.342, F (4,356) = 110.532, *p* < 0.01. SI also has a moderate and positive linear relationship with ESL teachers' BI to use technology after the COVID-19 pandemic, r (360) = 0.585, *p* < 0.01. This implies that ESL teachers are keen to adopt technology if the people around them persuade them to employ it in their ESL classroom.

#### 3.2.4. Hypothesis 4: There is no significant relationship between FC and Malaysian ESL teachers' BI to use technology in the post-COVID-19 era

Hypothesis 4 is not rejected, as FC is not a significant predictor of ESL teachers' BI to use technology in the post-COVID-19 era [β = 0.055, t (360) = 1.484, *p* > 0.05]. Moreover, the Pearson correlation test reveals a negligible relationship between FC and BI to use technology after the pandemic, r (360) = 0.283, p < 0.01. FC explains only 8% of the variance in BI [R^2^ = 0.080, F (4,356) = 110.532, *p* < 0.01], leading to failure to reject the null hypothesis in Hypothesis 4.

### 3.3. Research question 3: What is the most significant factor influencing Malaysian ESL teachers' behavioral intentions to use technology in the post-COVID-19 era?

In relation to the third objective of this study, the factor with the largest absolute value for the standardized coefficient (β), as shown in Table 3, represents the most significant factor in affecting teachers' intentions.

The results show that PE, EE, and SI are important in determining Malaysian ESL teachers' intentions. However, EE has the largest standardized coefficient (β = 0.325), implying that EE is the most significant factor. This variable is statistically significant at a level below the 0.01 level.

## 4. Discussion

### 4.1. Malaysian ESL teachers' technology acceptance level in the post-COVID-19 era

In relation to the first research question, Malaysian ESL teachers hold a high level of BI for the use of technology in the post-COVID-19 era, as BI had a mean and standard deviation of 4.32 and 0.669, respectively, with this mean value falling within the high-level range. Therefore, their high level of BI for technology use directly impacts their actual use of technology (Mohammad-Salehi et al., 2021; Kim and Lee, 2022; Sharma and Saini, 2022).

At the beginning of the pandemic, most Malaysian ESL teachers were not ready to implement ERT during the COVID-19 disruptions to regular classroom instruction. However, this lack of readiness did not affect their level of BI to integrate technology in the delivery of ESL lessons in the post-COVID-19 era. Based on the questionnaire findings, ~80% of the respondents intended to use technology for their future ESL teaching. Moreover, 84.2% of the respondents indicated that the COVID-19 pandemic had made them more inclined to employ technology more often when performing daily tasks in the future (Wen and Tan, 2020). This result is consistent with the findings of several researchers pertaining to relevant studies that have suggested that Malaysian ESL teachers tend to employ technology voluntarily in their daily working routines (Hu and AlSaqqaf, 2021; Omar and Hashim, 2021; Ting and Aziz, 2021; Siang and Mohamad, 2022).

In addition, this study has also identified the fact that most Malaysian ESL teachers express a willingness to adopt technology for use in various tasks that can improve their productivity. These tasks include the development of teaching aids (*n* = 323, 89.5%) and lesson delivery (*n* = 322, 89.2%). This finding parallels Li's study (2022), which found that teachers are willing to acquire technological knowledge and skills to integrate technology for instructional purposes. These findings suggest that Malaysian ESL teachers are accepting of technology and are willing to integrate it into their classrooms if they possess the requisite fundamental digital technology skills (Sari et al., 2021). Therefore, educational stakeholders should provide relevant training courses and programs to ESL teachers to develop their competency in ICT and apply the knowledge acquired in their respective ESL classrooms.

### 4.2. Relationships between the model factors and Malaysian ESL teachers' behavioral intentions for technology use in the post-COVID-19 era

The correlation results indicated that all factors in the UTAUT model, namely PE, EE, SI, and FC, are significantly correlated with respondents' BI to use technology in their ESL classrooms in future. Nevertheless, the result of the multiple regression analysis showed that their BI has a significant relationship only with PE, EE, and SI, whereas the factor of FC has no significant relationship with their BI to use technology in the ESL classroom during the post-COVID-19 era.

PE is identified as one of the factors that impact Malaysian ESL teachers' BI for using technology after the COVID-19 pandemic. In other words, teachers are concerned about whether technology can enhance the efficiency of their performance in their job (Yang et al., 2023). This finding is consistent with past studies, which also found an increase in teachers' BI to adopt technology if they found it useful and beneficial for them (Bajaj et al., 2021; Huang et al., 2021; Rashid et al., 2021; Ateş and Garzón, 2022; Mukminin et al., 2022; Khlaif et al., 2023). Relating the literature to the findings of the current study, Malaysian ESL teachers were exposed to technology in a way that they had never experienced before, enabling them to deliver ESL lessons at an optimum level. They utilized various websites and applications for lesson planning, for the preparation of teaching aids, and also for lesson delivery. Therefore, there is a strong possibility that Malaysian ESL teachers found technology to be effective in delivering lesson content during the COVID-19 pandemic (Al-Anezi and Alajmi, 2021; Mohammad-Salehi et al., 2021; Ting and Aziz, 2021), raising their BI to integrate technology for instructional delivery in their upcoming ESL lessons; this is contradictory to the findings of a few other studies (Sharma and Saini, 2022; Utami et al., 2022).

Moreover, EE is also vital in determining Malaysian ESL teachers' BI to adopt technology after experiencing ERT in conjunction with the COVID-19 pandemic. One of the most important factors for teachers to consider is the extent to which it is easy to use technology (Venkatesh et al., 2003). According to the findings of this study, they are more likely to have the intention of using technology if they find it user-friendly, as found in previous studies (Asghar et al., 2021; Dindar et al., 2021; Luik and Taimalu, 2021; Menabò et al., 2021; Rashid et al., 2021; Saidu and Mamun, 2022; Sharma and Saini, 2022). It is undeniable that most of them had to rely primarily on their digital devices throughout ERT during the pandemic. Accordingly, they were able to develop fundamental skills in handling mobile devices and digital applications. They were keen to continue using these for ESL instruction after the pandemic, as they found them easy to use and convenient (Gurer, 2021; Ting and Aziz, 2021).

Furthermore, the findings also demonstrate a significant relationship between the factor of SI and Malaysian ESL teachers' BI to employ technology in their post-COVID-19-era ESL classrooms. This indicates that they have greater intentions of using technological tools or applications if the people around them perceive this as beneficial or do the same. With reference to the questionnaire findings, the majority of Malaysian ESL teachers were influenced by their administrators, followed by peer teachers and colleagues. These findings are consistent with past studies, which have found that individuals close to teachers, such as colleagues, peers, and family members, were highly likely to engage with teachers and motivate them to use technology in implementing ERT during the COVID-19 pandemic (Asghar et al., 2021; Mohammad-Salehi et al., 2021; Rahman et al., 2021; Ting and Aziz, 2021; Jalil et al., 2022; Utami et al., 2022). Thus, in this study, it can be concluded that Malaysian ESL teachers receive positive influence and support from the people around them, resulting in their high level of BI to adopt technology in their work routines in the post-COVID-19 era.

In contrast, the results showed that the factor of FC does not have a significant relationship with Malaysian ESL teachers' intentions of adopting technology. Technology users' intentions to use technology will develop if they believe that they have access to the resources and tools needed to carry out their daily tasks (Wah and Hashim, 2021). Nevertheless, this study's findings indicated otherwise, showing that teachers' level of intention to use technology is not affected by the technological tools and resources at their disposal. This phenomenon could be explained using the findings obtained from the questionnaire. The descriptive analysis indicated that more than half of the respondents were doubtful or expressed disagreement that they had adequate technological equipment and resources, including Internet connection and technical assistance, resulting in a weak positive correlation and an insignificant relationship between FC and their intentions to use technology after the pandemic as testified by Aina and Opeyemi (2020). Although this finding failed to conform to the results presented in the majority of recent studies, there are also a number of studies that have reported similar results. For instance, Luik and Taimalu's study (2022) elucidated the fact that various technical challenges, such as a lack of technological devices and support, had impacted Nigerian teachers' ability to conduct online teaching, resulting in a lower level of technology acceptance. Similarly, some studies have reported that the presence of FCs has no effect on a technology user's BI to use technology systems due to an unsatisfactory level of FCs among users (Ramllah, 2020).

Taking all the findings into account, the conclusion of this study is that, of the four null hypotheses proposed, three were rejected; specifically, the factors of PE, EE, and SI were found to have significant relationships with Malaysian ESL teachers' BI to use technology in the post-COVID-19 era. In contrast, despite the discovery of a weak positive correlation between FC among Malaysian ESL teachers and their BI to use technology, the relationship between these variables is insignificant, resulting in failure to reject the null hypothesis. Due attention should be paid to this issue by educational stakeholders, as Malaysian ESL teachers could be encountering various constraints, such as a lack of access to sufficient technological facilities and devices to conduct ESL lessons, resulting in their reluctance to adopt technology in their respective ESL classrooms. In particular, a needs analysis should be conducted in future studies to identify Malaysian ESL teachers' requirements to provide quality ESL lessons.

### 4.3. The most significant factor affecting Malaysian ESL teachers' behavioral intentions for technology use in the post-COVID-19 era

Of the four factors discussed on the basis of the UTAUT model, PE, EE, and SI had a significant relationship with Malaysian ESL teachers' BI to use technology in their post-COVID-19-era ESL classrooms. However, EE emerged as the most significant factor affecting their BI, which suggests that the majority of Malaysian ESL teachers are more likely to have strong intention of integrating technology into their ESL classroom if they perceive doing so to be easy and effortless, as presented in previous studies (Asghar et al., 2021; Dindar et al., 2021; Luik and Taimalu, 2021; Menabò et al., 2021; Rashid et al., 2021; Saidu and Mamun, 2022; Sharma and Saini, 2022). If they find technology to be user-friendly, they will be accepting of it and attempt to implement it in their daily English lessons, resulting in positive development of their attitudes (Wah and Hashim, 2021). This can be explained by the amount of training they have previously received. Malaysian ESL teachers received adequate online training and attended multiple webinars to equip themselves with the relevant skills to use these online applications (Wen and Tan, 2020). Consequently, their digital competency level and perceptions of technology developed positively during the COVID-19 pandemic. Thus, they found technology to be simple and easy to use (Al-Anezi and Alajmi, 2021).

## 5. Conclusion

This study has achieved its objectives, as both ESL teachers' technology acceptance levels and factors affecting their behavioral intentions for technology use were identified. ESL teachers generally have a high level of technology acceptance, having embraced technology as one of the inevitable components of their work routine after being immersed in an ERT environment that relied on heavy use of technology throughout the pandemic. Consequently, they demonstrate a high degree of willingness to adopt technology in their post-COVID-19-era ESL classrooms. Moreover, PE, EE, and SI play vital roles in influencing teachers' BI to use technology in their ESL teaching in the post-COVID-19 era. This finding indicates that they are more likely to adopt technology in their ESL classrooms if they find it useful and easy to use, or if they are encouraged to do so by the individuals around them. Specifically, EE is the most significant factor, indicating that teachers are keen to use technology in the future if they find it effortless and uncomplicated to apply in their ESL classrooms.

This study constitutes an important contribution to the educational psychology literature, as it sheds light on the extent to which ESL teachers' behavioral intentions for technology use will be affected by the factors described in the UTAUT model; in particular, the findings demonstrate the changes in technology acceptance levels that have occurred due to the implementation of emergency remote teaching in dealing with the crisis of the COVID-19 pandemic. This study provides added value and insights for curriculum stakeholders and planners in terms of ways to encourage more ESL teachers to accept and employ technology to optimize the quality of their teaching. As this study found an insignificant relationship between facilitating conditions and ESL teachers' behavioral intention, educational stakeholders should investigate this issue further and take necessary actions to develop infrastructure, policy, instructional strategies, and design to enhance the acceptance and actual use of technology among ESL teachers.

For researchers who intend to further explore this topic, there are some suggestions that should be considered. Longitudinal research can be conducted to compensate for the drawbacks of the cross-sectional design adopted in this study. Moreover, future studies should attempt to widen the range of possible explanatory variables and include supplementary moderating or intervening variables from the UTAUT model and other technology acceptance models, such as the Technology Acceptance Model (TAM) and Theory of Planned Behavior (TPB). Future studies could also compare technology acceptance between different demographic backgrounds, a line of investigation that is lacking in this study. It is feasible to explore how cultural and contextual factors contribute to ESL teachers' behavioral intentions of integrating technology in their ESL classrooms.

## Data availability statement

The original contributions presented in the study are included in the article/Supplementary material, further inquiries can be directed to the corresponding authors.

## Ethics statement

Ethical review and approval was not required for the study on human participants in accordance with the local legislation and institutional requirements. The patients/participants provided their written informed consent to participate in this study.

## Author contributions

TWC and MMY conceived the study, participated in its design and coordination, performed the final analyses, and co-drafted the manuscript. TWC collected field data, entered study data, and assisted in the data analysis and interpretation of study results. Both authors read, revised, and approved the final manuscript.
