# Supplementary material for: Factors affecting Malaysian ESL teachers' behavioral intentions for technology use in the post-COVID-19 era
Source: Front Psychol. 2023 Mar 22;14:1127272. doi: 10.3389/fpsyg.2023.1127272 (PMC10074589; doi:10.3389/fpsyg.2023.1127272)
Supplement: Supplementary file 1 [file Table_1.docx]

Supplementary Material

Factors affecting Malaysian ESL teachers’ behavioral intentions for technology use in the post-COVID-19 era

Teo Woon Chun†, Melor Md Yunus†

**† Correspondence:** Teo Woon Chun, Melor Md Yunus
g-13430968@moe-dl.edu.my, melor@ukm.edu.my

**Supplementary Table 1.** Demographic profile.

|  |  |  | **Frequency, *n*** | **Percentage, %** |
| --- | --- | --- | --- | --- |
|  |  |  |  |  |
| Gender | Male |  | 61 | 16.9 |
|  | Female |  | 300 | 83.1 |
|  |  | Total | 361 | 100.0 |
|  |  |  |  |  |
| Age | 21 to 30 years |  | 112 | 31.0 |
|  | 31 to 40 years |  | 136 | 37.7 |
|  | 41 to 50 years |  | 69 | 19.1 |
|  | 51 to 60 years |  | 42 | 11.6 |
|  | 61 years and above |  | 2 | 0.6 |
|  |  | Total | 361 | 100.0 |
|  |  |  |  |  |
| State | Perlis |  | 1 | 0.3 |
|  | Kedah |  | 15 | 4.2 |
|  | Terengganu |  | 8 | 2.2 |
|  | Pahang |  | 18 | 5.0 |
|  | Perak |  | 43 | 11.9 |
|  | Kelantan |  | 8 | 2.2 |
|  | Penang |  | 14 | 3.9 |
|  | Selangor |  | 48 | 13.3 |
|  | Negeri Sembilan |  | 18 | 5.0 |
|  | Johor |  | 96 | 26.6 |
|  | Malacca |  | 11 | 3.0 |
|  | Kuala Lumpur |  | 11 | 3.0 |
|  | Sabah |  | 21 | 5.8 |
|  | Sarawak |  | 48 | 13.3 |
|  | Labuan |  | 1 | 0.3 |
|  |  | Total | 361 | 100.0 |
|  |  |  |  |  |
| Highest academic | SPM or equivalent |  | 0 | 0.0 |
| qualification | STPM or equivalent |  | 1 | 0.3 |
|  | Diploma |  | 12 | 3.3 |
|  | Bachelor’s Degree |  | 276 | 76.5 |
|  | Master’s Degree |  | 69 | 19.1 |
|  | Doctorate Degree |  | 3 | 0.8 |
|  |  | Total | 361 | 100.0 |
|  |  |  |  |  |
| Major discipline | TESL |  | 226 | 62.6 |
|  | English Studies |  | 28 | 7.8 |
|  | Others |  | 107 | 29.6 |
|  |  | Total | 361 | 100.0 |
|  |  |  |  |  |
|  |  |  |  |  |
| Experience of | 0 to 10 years |  | 241 | 66.8 |
| teaching English | 11 to 20 years |  | 75 | 20.8 |
|  | 21 to 30 years |  | 31 | 8.6 |
|  | 31 to 40 years |  | 14 | 3.9 |
|  |  | Total | 361 | 100.0 |
|  |  |  |  |  |
| Type of school | Government primary school |  | 293 | 81.2 |
|  | Government secondary school |  | 57 | 15.8 |
|  | Private primary school |  | 5 | 1.4 |
|  | Private secondary school |  | 6 | 1.7 |
|  |  | Total | 361 | 100.0 |
|  |  |  |  |  |
| Location of | Urban |  | 168 | 46.5 |
| school | Suburban |  | 104 | 28.8 |
|  | Rural |  | 89 | 24.7 |
|  |  | Total | 361 | 100.0 |
|  |  |  |  |  |

**Supplementary Table 2.** Findings of the study.

|  | **Strongly Disagree** | **Disagree** | **Neutral** | **Agree** | **Strongly Agree** |
| --- | --- | --- | --- | --- | --- |
| **Performance Expectancy (PE) (adapted from Mohammad-Salehi et al., 2021)** | | | | | |
| 1. I find technology useful in my ESL teaching instructions. | 0 | 1  (0.3%) | 23 (6.4%) | 125 (34.6%) | 212 (58.7%) |
| 1. Using technology enables me to accomplish ESL teaching goals more quickly. | 0 | 5  (1.4%) | 33 (9.1%) | 138 (38.2%) | 185 (51.2%) |
| 1. Using technology increases my productivity in ESL teaching instructions. | 0 | 4  (1.1%) | 33 (9.1%) | 141 (39.1%) | 183 (50.7%) |
| 1. Using technology enables my ESL learners to understand the content better. | 0 | 7  (1.9%) | 39 (10.8%) | 139 (38.5%) | 176 (48.8%) |
| **Effort Expectancy (EE) (Adapted from Huang et al. 2021)** | | | | | |
| 1. My interaction with technology for teaching ESL is clear and understandable. | 0 | 3  (0.8%) | 45 (12.5%) | 195 (54.0%) | 118 (32.7%) |
| 1. I find it easy to use technology to do what I want to do. | 0 | 7  (1.9%) | 44 (12.2%) | 148 (41.0%) | 162 (44.9%) |
| 1. I find technology easy to learn. | 3  (0.8%) | 11 (3.0%) | 59 (16.3%) | 158 (43.8%) | 130 (36.0%) |
| 1. I find it easy to be skilful at using technology. | 2  (0.6%) | 13 (3.6%) | 74 (20.5%) | 156 (43.2%) | 116 (32.1%) |
| **Social Influence (SI) (Ateş & Garzón, 2022; Mohammad-Salehi et al., 2021; Yunus et al., 2021)** | | | | | |
| 1. My family think that I should use technology for teaching ESL. | 2  (0.6%) | 14 (3.9%) | 100 (27.7%) | 137 (38.0%) | 108 (29.9%) |
| 1. My administrators think that I should use technology for teaching ESL. | 0 | 3  (0.8%) | 49 (13.6%) | 140 (38.8%) | 169 (46.8%) |
| 1. My colleagues think that I should use technology for teaching ESL. | 0 | 7  (1.9%) | 70 (19.4%) | 168 (46.5%) | 116 (32.1%) |
| 1. My peer teachers think that I should use technology for teaching ESL. | 0 | 6  (1.7%) | 58 (16.1%) | 171 (47.4%) | 126 (34.9%) |
| **Facilitating Conditions (FC) (Adapted from Thompson et al., 1991; Yunus et al., 2021)** | | | | | |
| 1. There is enough technology in the place where I work (school and home). | 16  (4.4%) | 66 (18.3%) | 94 (26.0%) | 129 (35.7%) | 56 (15.5%) |
| 1. The technology equipment and resources at the place where I work are adequate. | 21  (5.8%) | 62 (17.2%) | 111 (30.7%) | 110 (30.5%) | 57 (15.8%) |
| 1. The speed of the Internet connection at my workplace is adequate for my teaching | 43 (11.9%) | 77 (21.3%) | 110 (30.5%) | 85 (23.5%) | 46 (12.7%) |
| 1. When I encounter technical difficulties, specialised help is available to me. | 39 (10.8%) | 74 (20.5%) | 115 (31.9%) | 94 (26.0%) | 39 (10.8%) |
| **Behavioural intention (BI) (Adapted from Mohammad-Salehi et al., 2021)** | | | | | |
| 1. I intend to use technology in my future ESL teaching. | 0 | 6  (1.7%) | 37 (10.2%) | 141 (39.1%) | 177 (49.0%) |
| 1. I intend to use technology to find or produce teaching aids for my ESL learners. | 0 | 3  (0.8%) | 35 (9.7%) | 148 (41.0%) | 175 (48.5%) |
| 1. I intend to use technology to deliver the content to my ESL learners. | 0 | 7  (1.9%) | 32 (8.9%) | 151 (41.8%) | 171 (47.4%) |
| 1. I intend to use technology more often compared to before the COVID-19 pandemic. | 5  (1.4%) | 14 (3.9%) | 38 (10.5%) | 147 (40.7%) | 157 (43.5%) |
